# Supplementary material for: Unravelling the Distinct Phenotype and Mechanosensitive Properties of Different Tendon Cell Populations
Source: Adv Sci (Weinh). 2026 Sep 13:e77391. Online ahead of print. doi: 10.1002/advs.77391 (PMC13572792; doi:10.1002/advs.77391)
Supplement: Supplementary file 1 [file ADVS-9999-e77391-s001.doc]

***Figure S1: Timeline for cell phenotype drifting analysis.*** *A schematic of the experimental protocol identifies the analysis time points selected for exploring IFM and FM cell phenotype drift though passage on TCP.* *Schematic created with BioRender.com.*

***Figure S2. IFM cells show a progressive decline in proliferative capacity during serial passaging on tissue culture plastic****. (A) Percentage increase in IFM and FM cell numbers between day 0 and day 6 at each passage, determined by haemocytometer cell counts. IFM and FM cells proliferated at a similar rate at P0, whereas IFM cell expansion progressively declined with increasing passage number. (B) Cumulative population doubling level (cPDL), calculated from the haemocytometer cell count data shown in (A) using the standard population doubling level equation (PDL = log₂(Nf/Ni)). IFM cells accumulated significantly fewer population doublings than FM cells during serial passaging, consistent with their reduced proliferative capacity on tissue culture plastic.* *Statistical significance for both (A) and (B) was assessed using a one-way ANOVA followed by Tukey's post-hoc test within each cell type (mean ± SD, n = 4 rats; *P < 0.05, **P < 0.01).*

***Figure S3: Timeline for cell phenotype recovery analysis.*** *a) Schematic illustration of the protocol in which cells were cultured on TCP until either P1 or P3 at which point they were transferred to a range of softer substrates to explore the recovery of cell phenotype. b) The time points and passages selected for each analysis approach are outlined.* *Schematic created with BioRender.com.*

***Figure S4: Compressive stress-strain behaviour of different PDMS concentrations.*** *Compressive stress-strain curves for PDMS samples with varying compositions of PDMS 527 and PDMS 184. Samples tested include PDMS 527/PDMS 184 ratios of 1:20, 3:20, 8:20, and pure PDMS 184, with n=6 for each composition. The Young’s modulus for each sample was calculated from the gradient of the linear region of the stress-strain curve between 0–10% compression (shown in yellow).*

***Figure S5: IFM cell proliferation recovers on softer surfaces.*** *DNA quantification for cells cultured for 4 days across the range of stiffness highlights that IFM cell proliferation is higher on softer surfaces, and consistent whether cells are transferred at P1 (top row) or P3 (bottom row), whilst FM cell proliferation is unaffected by TCP culture or transfer to softer surfaces* (*n = 4 rats). DNA quantity is measured using Hoechst and normalised to day 1 (TCP) for each individual experiment.* *Statistical significance was assessed using a two-way ANOVA followed by Tukey's post-hoc test. * Highlights significant differences across days, $ highlights a significant difference in a stiffness condition relative to TCP. * or $ = p<0.05, ** or $$ = p< 0.01, *** or $$$ = p<0.001, **** or $$$$ = p<0.0001.*

**Figure S6:** **Morphological characteristics of IFM and FM cells in culture on TCP and PDMS substrates over passages.** IFM-FM cell morphology parameters on varying substrate stiffnesses, showing cell body area, nucleus area, actin abundance/cell, cell eccentricity, and cell alignment, for IFM cells (green) and FM cells (red). Data is presented using the Kernel Density Estimation (KDE) method, with the right side of each violin plot representing the distribution and the left side showing the dataset repartition, for a minimum of 200 cells in each condition with error bars indicating standard error (n = 4 rats). Data on the left (blue background) depicts response from P0 to P2 when cells were cultured across passages on TCP. Data on the right (yellow background) depicts cell response when transferred onto a range of increasing softer substrates (1.5 MPa to 4–8 kPa) at the start of P3. Within each condition, day 2 of culture is shown in light green or light red and day 4 in dark green or dark red. Day 4 is missing in most FM cell conditions as cells were too confluent to accurately assess parameters. Day 2 at P0 is missing as cells were still settling. Statistical significance was assessed using a two-way ANOVA followed by Tukey's post-hoc test. Statistical significance relative to the P0 TCP reference is indicated by * = p < 0.001. Data is summarised in figure 7a.

***Figure S7: IFM cell morphology is strongly impacted by substrate stiffness.*** *Correlations between cell body area, nucleus area and actin abundance/cell are shown for IFM cells (green) and FM cells (red) with the associated Pearson corelation coefficient (r) and the slope (m).*

**Figure S8:** **Heatmap of average gene expression values for the proposed IFM cell and FM cell clusters derived from equine SDFT single-cell RNA sequencing** (8)**.** A calculation of the relative abundance of IFM and FM genes from young equine single cell RNA sequencing data (n=4), calculated using the same method as adopted for the analysis of isolated rat IFM and FM cells in the current study (Figure 2) to facilitate comparison. Values represent the log₂ fold change of gene expression in IFM relative to FM, normalised to GAPDH. A paired t-test was performed for each gene (*= p<0.05, **= p< 0.01, ***=p<0.001, ****= p<0.0001).

| **Antibody** | **Concentration** | **Product code** |
| --- | --- | --- |
| Anti-Scx A | 1/100 | Abcam Ab58655 |
| Anti-CD146 | 1/100 | Abcam Ab75769 |
| Anti- TPPP3 | 1/100 | Abcam Ab150998 |
| Anti- tnmd | 1/100 | Abcam Ab203676 |
| Anti-LAMA4 | 1/100 | St John’s Laboratory  STJ93891 |
| Alexa Fluor 647 goat anti-rabbit | 4 µug/mL | Thermofisher Scientist  a21244 |

***Figure S9:*** ***List of antibodies, concentrations and product code specifications used for immunofluorescence on rat tail tendons.***
